# Multi-Object Detection of Forage Density and Dairy Cow Feeding Behavior Based on an Improved YOLOv10 Model for Smart Pasture Applications

**DOI:** 10.3390/s26041273

**Published:** 2026-02-15

**Authors:** Zhiwei Liu, Jiandong Fang, Yudong Zhao

**Affiliations:** 1 College of Information Engineering, Inner Mongolia University of Technology, Hohhot 010080, China; 17678001689@163.com; 2 Inner Mongolia Key Laboratory of Intelligent Perception and System Engineering, Hohhot 010080, China; 3 Inner Mongolia Synergy Innovation Center of Perception Technology in Intelligent Agriculture and Animal Husbandry, Hohhot 010080, China

**Keywords:** dairy cattle feeding behavior, forage density detection, YOLOv10, feature fusion

## Abstract

In modern smart dairy farms, precise feed management and accurate monitoring of dairy cows’ feeding behavior are crucial for improving production efficiency and reducing feeding costs. However, in practical applications, complex environmental factors such as varying illumination, frequent occlusion, and dense multi-targets pose significant challenges to real-time visual perception. To address these issues, this paper proposes a lightweight multi-target detection model, BFDet-YOLO, for the joint detection of dairy cows’ feeding behavior and feed density levels in pasture environments. Based on the YOLOv10 framework, the model incorporates four targeted improvements: (1) a bidirectional feature fusion network (BiFPN) to address the insufficient multi-scale feature interaction between dairy cows (large targets) and feed particles (small targets); (2) a lightweight downsampling module (Adown) to preserve fine-grained features of feed particles and reduce the risk of small target miss detection; (3) an attention-enhanced detection head (SEAM) to mitigate occlusion interference caused by cow stacking and feed accumulation; (4) an improved bounding box regression loss function (DIoU) to optimize the localization accuracy of non-overlapping small targets. Additionally, this paper constructs a pasture-specific dataset integrating dairy cows’ feeding behavior and feed distribution information, which is annotated and expanded by combining public datasets with on-site monitoring data. Experimental results demonstrate that BFDet-YOLO outperforms the original YOLOv10 and other mainstream target recognition models in terms of detection accuracy and robustness while maintaining a significantly streamlined model scale. On the constructed dataset, the model achieves 95.7% mAP@0.5 and 70.7% mAP@0.5:0.95 with only 1.85 M parameters. These results validate the effectiveness and deployability of the proposed method, providing a reliable visual perception solution for intelligent feeding systems and smart pasture management.

## 1. Introduction

In modern smart livestock farming, precise feeding and scientific feed management are key determinants in enhancing pasture productivity. Optimizing feed allocation and utilization not only reduces farming costs but also improves dairy cow health and increases milk yield [1]. The essence of precision feeding lies in achieving the dynamic matching between “dairy cows’ feeding demands” and “forage supply status”, and the key prerequisite for this matching is the accurate measurement of forage density and cows’ feeding behavior. From the perspective of production practice, the real-time measurement of forage density serves as the core basis for optimizing feeding strategies: excessively low density is prone to insufficient feeding for cows, affecting their health and production performance; excessively high density may lead to forage waste and increased costs, while an appropriate density is crucial for balancing feeding efficiency and cost control. Meanwhile, cows’ feeding behavior is a direct signal reflecting their feeding demands, and its distribution and changes can guide the priority allocation of feeding resources: forage supply needs to be maintained in areas with intensive feeding, feeding planning should be advanced in areas where cows are “approaching”, and operations in areas without relevant behaviors can be postponed. However, most pastures in China still rely on manual feeding or fixed-path feed pushing, which entails high labor intensity, low operational efficiency, and significant feed waste, struggle to meet the requirements of refined and intelligent management in modern smart pastures [2]. In large-scale farming scenarios, forage pushing operations must cope with complex and dynamic environmental conditions as well as cows’ variable feeding behaviors, making the construction of an efficient automated forage pushing system and optimal forage resource allocation a critical challenge for pasture intelligence upgrades.

In recent years, with the rapid development of artificial intelligence, the Internet of Things, and edge computing technologies, intelligent forage pushing robots have gradually become an integral part of smart pastures [3]. Compared to traditional mechanical equipment, the core advantage of intelligent forage pushing systems lies in their ability to dynamically adjust feeding strategies based on environmental perception, achieving more precise and efficient forage management. However, existing forage pushing robotic systems mostly rely on fixed patrol modes or simple sensor feedback, and their performance bottleneck does not stem from the actuators themselves but rather from the lack of reliable perception of forage distribution and cows’ feeding behavior in pasture environments [4]. In complex pasture environments, factors such as lighting variations, forage morphology differences, cow occlusion, and the coexistence of multiple targets further increase the difficulty of perception tasks, making the development of high-precision, real-time visual perception models a key prerequisite for the practical deployment of intelligent forage pushing systems.

Under the Livestock 4.0 paradigm, computer vision-based behavior recognition technologies have been widely applied in animal health monitoring and behavior analysis. Among these, object detection algorithms have demonstrated strong potential in multi-target behavior recognition tasks due to their end-to-end nature, high efficiency, and scalability [5]. As a representative single-stage object detection approach, the YOLO (You Only Look Once) series achieves a good balance between detection accuracy and real-time performance and has been successfully applied to behavior monitoring of pigs, poultry, and cattle, including dairy cow feeding behavior recognition [6,7,8,9,10], estrus detection [11,12], and individual tracking analysis [13,14]. These studies indicate that YOLO models are robust and generalizable in complex livestock scenarios, particularly suitable for pasture environments requiring real-time monitoring.

Beyond animal behavior recognition, forage state perception is also a fundamental basis for precise feeding. Previous studies in aquaculture have explored YOLO-based forage recognition methods for detecting uneaten pellet forage and have achieved good detection performance [15,16]. However, compared with aquaculture settings, pasture environments present more complexity in forage morphology, background textures, lighting conditions, and occlusions, making direct transfer of existing methods challenging. Relative to generic object detection paradigms, the joint detection of dairy cattle feeding behavior and forage density grading within pasture environments confronts a distinctive constellation of exclusive, multifaceted challenges. Whereas conventional detection frameworks predominantly address scenarios characterized by structured backgrounds and salient target features—typically operating through single-category independent detection—pastoral forage exhibits amorphous configurations with indistinct boundary characteristics, necessitating fine-grained hierarchical grading rather than simplistic target localization. The dense stacking of multiple heads during cattle feeding episodes, compounded by mutual occlusions between livestock bodies and forage, precipitates incomplete target feature representations; such dynamic occlusion issues manifest with substantially greater frequency than in generic detection scenarios. Furthermore, uncontrolled environmental variables—including diurnal photometric fluctuations, background clutter interference, and chromatic feature ambiguity between cattle and forage—considerably exacerbate the complexity of target recognition and segmentation. Concurrently, the dual-target joint detection paradigm necessitates accommodating multi-task requirements encompassing both behavioral judgment and density grading, thereby imposing heightened demands upon the model’s capabilities for feature coupling and weight allocation. Additionally, edge computing deployments for intelligent forage pushing operations impose rigid constraints upon real-time performance and architectural lightweighting, engendering a stringent “accuracy–speed–resource” trilemma. Currently, research on forage density grading detection in pasture environments remains limited, particularly lacking unified frameworks that consider both forage distribution and cow feeding behavior. Moreover, most existing studies focus on individual cows or behavior recognition itself, with insufficient attention to fine-grained forage state perception, limiting the practical applicability of visual perception results for intelligent feeding decisions. Although other computer vision algorithms such as ResNet, Faster R-CNN, and RetinaNet have been applied in animal science, YOLO achieves a favorable balance of accuracy, unified architecture, and flexibility. Importantly, its high speed and real-time capability are well aligned with on-site pasture requirements [17,18,19]. Among various YOLO versions, YOLOv10 introduces an end-to-end non-maximum suppression (NMS) training strategy and more efficient feature representation, significantly reducing model latency and computational cost while maintaining high detection accuracy, providing a promising solution for resource-constrained edge computing scenarios.

Feature fusion, serving as a pivotal technique for enhancing multi-object detection precision in complex scenarios, aims to alleviate the semantic discrepancy between deep semantic information and shallow spatial details through cross-layer feature interaction. Within the domain of intelligent agricultural detection, sophisticated feature fusion strategies have been empirically validated to significantly augment target perception capabilities under complex background conditions. Addressing challenges associated with variable target scales and background texture similarities in farmland residual plastic film detection, Zhang et al. [20] proposed FreqDyn-YOLO, constructing a High-Performance Multi-Scale Feature Pyramid Network (HPMSFPN) that employs cross-layer feature integration mechanisms coupled with Efficient Multi-scale Feature Convolution (EMFC) modules, thereby achieving efficient fusion of shallow detail features and deep semantic information, and effectively enhancing detection performance for low-contrast targets between residual films and soil backgrounds. Oriented toward broader unmanned aerial vehicle (UAV) aerial photography scenarios, Yan et al. [21] introduced DMF-YOLO, targeting challenges of substantial scale variations, small target proportions, and dense occlusions in drone imagery by designing Dilated Dynamic Serpentine Convolution (DDSConv) and Multi-scale Feature Aggregation Modules (MFAM); through dual-branch spatial attention mechanisms that adaptively weight critical spatial regions, this approach effectively resolved the inability of conventional PAN-FPN architectures to integrate high-order semantics with low-order spatial details, achieving high-precision small object detection in complex aerial environments encompassing emergency rescue and maritime surveillance. The aforementioned studies substantiate the efficacy of multi-scale feature fusion in complex environments; however, their application contexts predominantly involve static backgrounds or rigid targets, lacking adaptive designs for dynamic multi-object coupling scenarios characteristic of pasture environments. To further advance discriminative capacity for fine-grained targets, Xie et al. [22], addressing the issues of complex backgrounds and subtle target features in remote sensing imagery, proposed a feature fusion methodology based on discriminative representation learning; by intensifying the distinction between target critical features and background noise, this approach achieved precise detection of remote sensing fine-grained targets. The core innovation resides in excavating fine-grained target characteristics and accomplishing multi-scale fusion, effectively resolving the issue of target feature submersion in complex backgrounds. Nevertheless, the application context of this investigation comprises remote sensing image detection, with detection targets characterized as static, high-resolution artificial or natural features; while backgrounds exhibit complexity, they lack dynamic interaction characteristics. Moreover, targeted designs for model lightweighting and real-time performance remain absent, engendering fundamental divergences from the application requirements of pasture scenarios. Conversely, the feature fusion mechanism inherent to native YOLOv10 adopts conventional top-down concatenation approaches, exhibiting suboptimal efficiency in multi-scale feature interaction and demonstrating insufficient capacity for extracting and fusing weak forage edge features and incomplete features arising from cattle occlusions, thereby proving inadequate for the exclusive detection challenges of pasture environments and necessitating targeted modifications.

Based on the above analysis, this study proposes a lightweight multi-object detection model, BFDet-YOLO, tailored for pasture environments. Built upon YOLOv10, it enables joint detection of dairy cow feeding behavior and forage density grading. By introducing a bi-directional feature fusion structure, lightweight downsampling modules, an attention-enhanced detection head, and an improved bounding box regression loss function, the proposed method significantly improves detection accuracy and robustness while maintaining real-time performance. Experimental results demonstrate that the model outperforms the original YOLOv10 and several mainstream YOLO variants on a self-constructed pasture dataset, validating its effectiveness and practical value for visual perception and intelligent feeding in smart pastures.

The contributions of this work are as follows: Firstly, a multi-source integrated pasture experimental dataset was constructed, combining publicly available online resources with self-collected monitoring data. Through unified frame extraction, splitting, and annotation procedures, images containing cow behavior and forage distribution were acquired, and the two data components were fused and augmented to form a complete experimental dataset, providing rich and high-quality data support for subsequent smart pasture recognition and forage pushing research. Secondly, an improved YOLOv10-based object detection algorithm, BFDet-YOLO, is proposed, with the following key innovations:

1. Bi-directional Feature Fusion (BF): The original top-down feature concatenation mechanism is replaced with a BiFPN-style bidirectional fusion structure to enable efficient multi-scale feature interaction and enhance semantic information transfer across layers.
2. Lightweight Node Enhancement: C2f-based node structures replace traditional concatenation and convolution modules, reducing computational cost while improving feature representation capability.
3. Improved Detection Head (Det): A more efficient detection head is designed to better integrate the fused features, showing superior localization accuracy and classification robustness for small- and medium-sized targets.
4. Scalability and Efficiency: While maintaining the real-time inference advantage of YOLO, the overall detection accuracy is improved, making it suitable for resource-limited embedded and edge computing scenarios.

Experimental results show that BFDet-YOLO achieves higher detection accuracy on the dataset compared to the original YOLOv10, validating the effectiveness of the proposed method.

## 2. Materials and Methods

### 2.1. Dataset Construction and Data Augmentation

The dataset used in this study was constructed by integrating the publicly available CBVD-5 dairy cow behavior video dataset from Kaggle with a self-collected pasture dataset. The self-collected data were acquired at Shengqingyuan Pasture, Hohhot, Inner Mongolia, China. The CBVD-5 dataset contains dairy cow behavior videos captured by seven surveillance cameras equipped with 2.8 mm and 3.6 mm lenses (Zhejiang Dahua Technology Co., Ltd., Hangzhou, China). The data acquisition system employed a Dahua DH-S3000C-16GT 16-port Gigabit network switch (Zhejiang Dahua Technology Co., Ltd., Hangzhou, China) and a DH-NVR2216-HDS3 network video recorder (Zhejiang Dahua Technology Co., Ltd., Hangzhou, China), enabling 24-h continuous monitoring over five consecutive days. The dataset includes 687 video clips and multiple folders of extracted frames.

**Figure 1:**
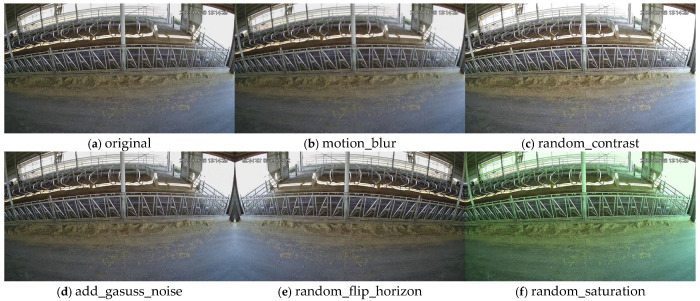
Image augmentation: (**a**) Original image, (**b**) Motion blur, (**c**) Randomly adjusted image contrast, (**d**) Added Gaussian noise, (**e**) Random horizontal flip, (**f**) Randomly adjusted image saturation.

From the CBVD-5 dataset and the self-collected dataset, a total of 1777 images containing dairy cow behavior and forage information were selected as the base dataset, supplemented by 194 images from the self-collected dataset. Data augmentation techniques were applied to approximately 15% of the images, including horizontal flipping, random contrast adjustment, random saturation adjustment, Gaussian noise injection, and motion blur simulation. Figure 1 shows the schematic diagram of data augmentation effects. Consequently, 1226 augmented images were generated, resulting in a final dataset of 3197 images that integrate both dairy cow behavior and forage density information.

**Figure 2:**
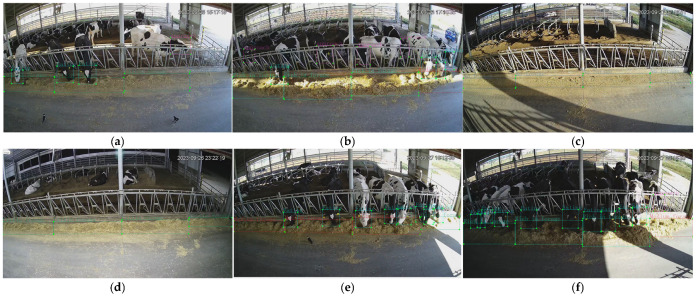
Examples of dataset annotations: (**a**) contains annotations for Eating, Approach, and No forage; (**b**) contains annotations for Eating, Approach, and Rich in forage; (**c**) contains annotations for No forage; (**d**) contains annotations for Little forage; (**e**) contains annotations for Eating and Medium forage; (**f**) contains annotations for Eating, Rich in forage, and Medium forage.

In this study, images were manually annotated based on expert knowledge and classified into the following categories: Eating, approach, no forage, Little forage, Medium forage, and Rich in forage. The annotation criteria are presented in Table 1. Annotation examples are shown in Figure 2.

Forage dataset annotation method: The feeding trough in the cow feeding area beneath the camera was divided into multiple regions, each 2.5 m in length. Forage within each region was annotated according to the dataset’s annotation criteria. Forage density was classified into four categories: No forage, Little forage, Medium forage, and Rich in forage, each corresponding to a different forage pushing strategy.

Cow dataset annotation method: The cow feeding area beneath the camera was divided into multiple sub-regions at 2.5-m intervals. Within each sub-region, two target bounding boxes (in YOLO format)—“cow head” and “forage region”—were first annotated independently. A cow was labeled as “Eating” if either of the following conditions was met: the Intersection over Union (IOU) of the two target bounding boxes was ≥0.1, or the y-coordinate of the lowest point of the cow head was ≤the y-coordinate of the highest point of the forage region, with the cow’s body distance from the feeding trough ≤ 1 body length. A cow was labeled as “approach” if its body distance from the feeding trough ≤ 1 body length, its head was protruding or about to protrude into the feeding trough, but the aforementioned eating determination conditions were not satisfied (IOU < 0.1 and the y-coordinate of the lowest point of the cow head > the y-coordinate of the highest point of the forage region), and there were no visual features of the mouth or nose making contact with the forage. Multiple cows in the same subregion and the same frame were annotated independently. The label file records each object in the format class_id x_center y_center width height (YOLO format), with all coordinates normalized to the range 0–1.

Finally, all the images were randomly divided into a training set, validation set and test set in a ratio of 6:2:2. The training set consists of 1918 images, while the validation set and test set contain 639 and 640 images respectively. Table 2 shows the distribution of different labels in the dataset.

### 2.2. Research Background and Methods

In large-scale pastures, uneven forage distribution directly leads to decreased forage intake, fluctuating milk production, and increased hoof diseases. Traditional manual forage pushing requires 2–3 round trips per day, which is labor-intensive and makes timely forage replenishment during early morning hours challenging. Although ground forage pushers already exist, they are limited by the “blind pushing” mode, which cannot perceive differences in forage levels, resulting in over-pushing and under-pushing. Simply installing on-board vision can lead to misjudgments due to dust and manure obstructions.

**Figure 3:**
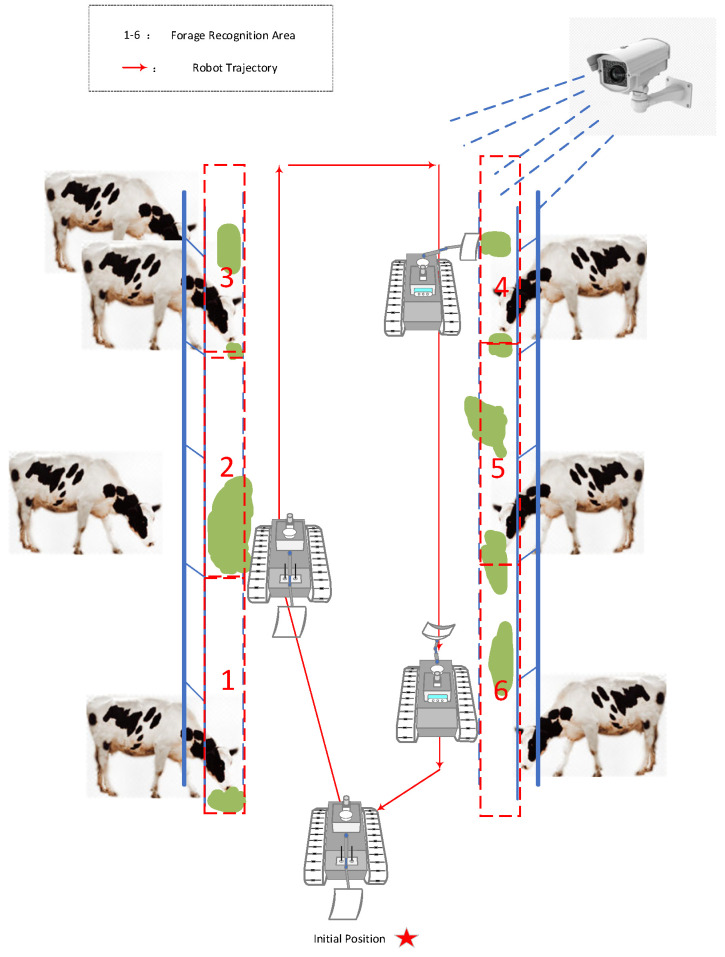
Schematic Diagram of Air-Ground Collaborative Forage Pushing.

Therefore, introducing an aerial perspective to achieve “see clearly before pushing” has become an urgent need for unmanned feeding in pastures. The air-ground collaborative forage pushing process is shown in Figure 3: Cameras deployed in the pasture (aerial perspective) monitor the forage levels in areas 1–6 globally, combined with the trajectory of the ground forage-pushing robot. First, aerial vision accurately identifies the forage level status in each area, then guides the robot to complete targeted forage pushing as needed, avoiding misjudgments due to single-view occlusions and solving the resource waste problem of “blind pushing.”

The YOLO algorithm is playing an increasingly important role in the development of agriculture and animal husbandry [23]. Since the introduction of the single-stage grid regression detection framework by YOLOv1 in 2015, the YOLO family has continuously broken through with the main line of “real-time + accuracy” [24]. YOLOv2 introduced BatchNorm and Anchor boxes to improve recall. YOLOv3 solved the problem of small target detection through Darknet-53 and FPN multi-scale fusion. YOLOv4 used CSPNet + PANet + self-developed Mosaic strategy to push the COCO mAP to 43% for the first time. YOLOv5 achieved adaptive anchor boxes and one-click export through engineering, with the nano version being only 1.9 M and able to run on Raspberry Pi [25]. Subsequently, YOLOv6/7/8/9 introduced technologies such as BiC, E-ELAN, C2f cross-stage fusion, and GELAN-PGI, continuously refreshing speed and accuracy records [26]. YOLOv10 achieved a 39.5% AP with only 2.3 M parameters and 6.7 GFLOPs through consistent double-label allocation without NMS, spatial-channel decoupled downsampling, and sorting-guided blocks, with a delay of only 1.84 ms [27]. This makes YOLOv10 the preferred detection engine for the multi-camera real-time forage pushing system in this paper’s smart pasture.

### 2.3. Improved YOLOv10

YOLOv10 has undergone structural streamlining and algorithmic enhancement based on YOLOv5 and YOLOv8: It continues to use the anchor-free framework but reduces the number of parameters by about 18% compared to YOLOv8 through the introduction of depthwise separable convolutions and channel-layer adaptive pruning [28]. It replaces YOLOv8’s C2F+ fixed FPN/PANet topology with a lightweight dynamic feature fusion module DyFPN, achieving adaptive weight reassignment of cross-level features [29]. On the classification side, it upgrades YOLOv8’s varifocal loss (VFL) to focal-varifocal hybrid loss (Focal-VFL), and on the regression side, it adds edge-aware loss (EAL) to CIoU + DFL, significantly alleviating the problem of missing small dense targets [30]. For the first time in the YOLO series, dynamic scale reasoning (DSR) is introduced to dynamically prune network depth based on input resolution, reducing the average latency on the edge side by 25% compared to YOLOv8 [31]. Anchor-free matching combined with label assignment smoothing is used to optimize the training convergence stability by eliminating the manually set thresholds in YOLOv5/v8 [31].

Nevertheless, existing foundational YOLO-based agricultural detection systems predominantly concentrate upon unitary targets, confronting scenario-specific challenges—including intermingled multi-scale targets, frequent occlusions, and environmental disturbances—within joint cattle–forage detection contexts where isolated enhancement modules prove inadequate to simultaneously satisfy the dual requisites of dairy cattle behavioral identification accuracy and forage density detection robustness. Accordingly, this investigation proposes the BFDet-YOLO architecture, achieving substantial performance breakthroughs in joint cattle–forage detection through synergistic design across the “feature fusion–downsampling–detection head–loss function” pipeline. The salient innovation resides in modular adaptation and integrative optimization tailored for pasture joint detection scenarios, rather than de novo conceptualization of discrete modules.

**Figure 4:**
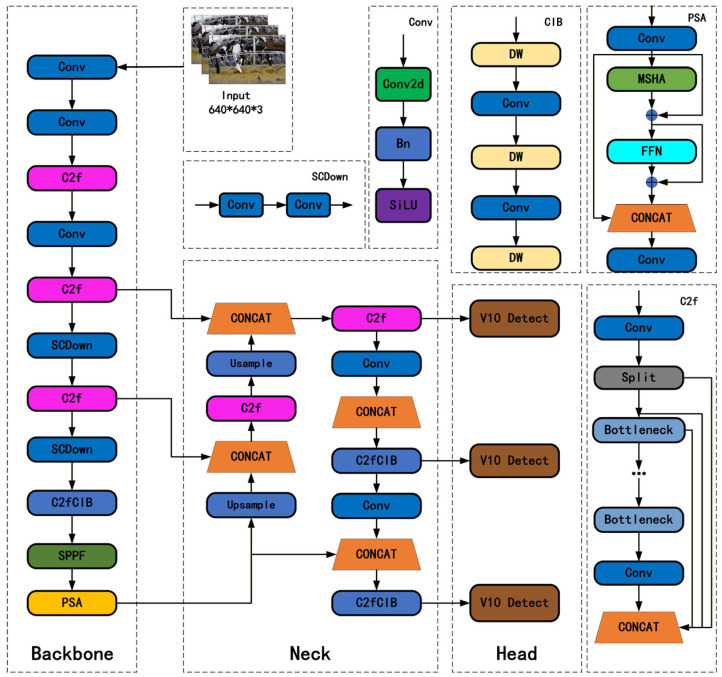
Original Network Structure of YOLOv10n.

**Figure 5:**
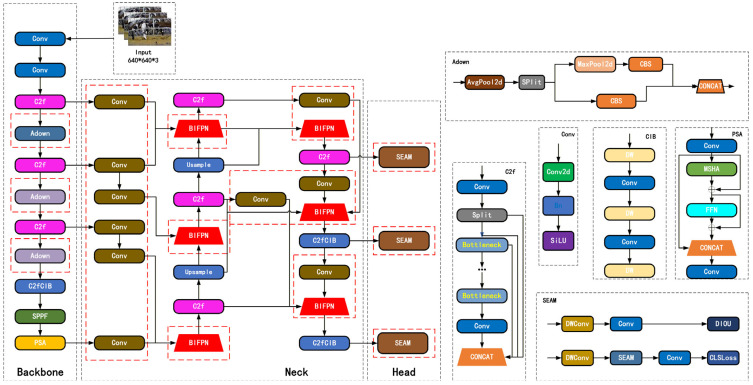
Network Structure of BFDet-YOLO.

BFDet-YOLO has made several improvements to the original YOLOv10 network: The backbone retains the original structure, including the C2f nodes, SPPF, and PSA modules. In the neck section, the original top-down feature fusion structure is replaced with a BiFPN-style bidirectional feature fusion (BF) to achieve efficient interaction of multi-scale features. Meanwhile, the original C2f node structure is enhanced for lightweighting to reduce computational load and improve feature representation. In the detection head, more efficient fusion and convolution modules are designed to better integrate the fused features, thereby improving the localization accuracy and classification robustness of small and medium-sized targets. Through these improvements, BFDet-YOLO significantly enhances detection capabilities while maintaining the real-time inference advantages of the YOLO series and achieving model lightweighting. Distinct from conventional YOLO-based agricultural detection paradigms, the present investigation establishes its core novelty through the elaboration of modular synergistic adaptation mechanisms specifically engineered for the distinctive scenario of coupled cattle–forage detection—characterized by the coexistence of bovine subjects and particulate forage substrates alongside the intricate interplay between static and kinetic targets—thereby transcending the rudimentary aggregation of pre-existing technological modules. Figure 4 shows the original YOLOv10 model, and the network structure of BFDet-YOLO is shown in Figure 5, with the improved parts highlighted in red.

#### 2.3.1. BiFPN-Style Bidirectional Feature Fusion (BF)

To enhance the multi-scale feature fusion capability of the model for dairy cow behavior and feed density recognition while maintaining its lightweight property, this study adopts Bidirectional Feature Pyramid Network (BiFPN) to replace the traditional Feature Pyramid Network (FPN) structure. Following the bidirectional path design of PANet, BiFPN constructs top-down (transmitting high-level semantic information) and bottom-up (transmitting low-level positional information) feature flows, and simultaneously introduces learnable weight parameters to achieve differentiated weighted fusion according to the contribution of features at different scales to the detection task. The specific fusion formula is as follows:

(1) $$F_{fusion}^{k}=\frac{\omega_{up}\cdot F_{up}^{k}}{\epsilon +\omega_{up}+\omega_{low}}$$

where $\omega_{up}$ and $\omega_{low}$ are the learnable weights of high-level semantic features and low-level positional features, respectively. $\epsilon =10^{-8}$ is used to avoid division by zero. During training, the model adaptively optimizes the weight proportion—in the scenario of dairy cow behavior recognition, $\omega_{up}$ is strengthened to highlight behavior-discriminating features, while in the scenario of forage density estimation, $\omega_{low}$ is enhanced to capture fine-grained distribution features.

**Figure 6:**
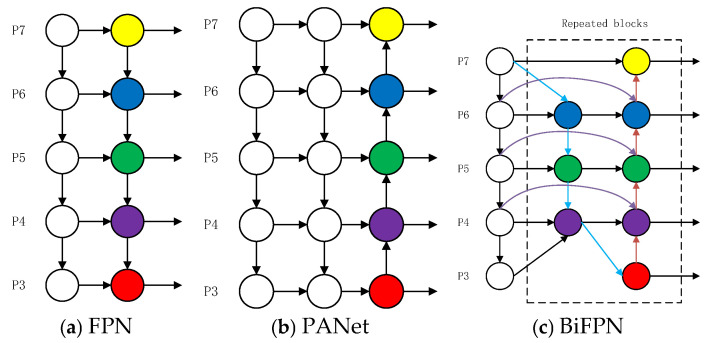
Feature Network Design: (**a**) FPN introduces a top-down path to fuse multi-scale features from level 3 to level 7 (P_3_–P_7_). (**b**) PANet adds an additional bottom-up path based on FPN. (**c**) BiFPN adopts bidirectional cross-scale connections, with the blue parts representing the top-down path that conveys semantic information of high-level features, the red parts representing the bottom-up path that conveys positional information of low-level features, and the purple parts indicating the newly added edges between input nodes of the same layer mentioned previously. This design achieves a better balance between accuracy and efficiency.

This design not only fully interacts high-level and low-level features through bidirectional cross-scale connections (Figure 6c) to strengthen the feature representation of key regions of dairy cow behavior and feed density details, but also controls the computational complexity by optimizing network redundancy. Compared with FPN with only a top-down path (Figure 6a) and PANet with an additional bottom-up path (Figure 6b), it achieves a better balance between accuracy and efficiency, meeting the dual requirements of model lightweight and high precision.

Figure 6 illustrates the network structure and bidirectional feature fusion mechanism of BiFPN, and compares the designs of different feature fusion networks.

#### 2.3.2. Target-Adaptive Design of Lightweight ADown Downsampling

**Figure 7:**
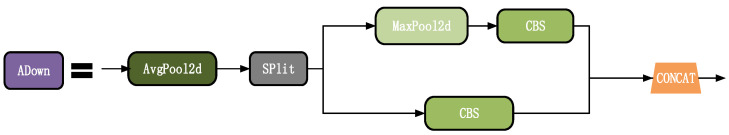
Structure Diagram of ADown Module.

To address the issue of small-target omission arising from overlapping forage blades and illumination fluctuations, whilst concurrently preserving the structural integrity of large targets corresponding to bovine bodies, this study substitutes specific CBS (Convolution-Batch Normalization-SiLU) modules within the YOLOv10n architecture with lightweight ADown downsampling units. As depicted in Figure 7, this module adopts an asymmetric “pooling-convolution” topology: average pooling operations retain holistic structural information of cattle bodies, whereas dual-pathway feature extraction—employing 3 × 3 convolutions to distill forage semantics and 3 × 3 max pooling to capture blade edges—targetedly enhances characteristics of diminutive targets. Diverging from generic ADown implementations, this work adjusts kernel parameters and channel allocation ratios to ensure that the module, during the downsampling process, neither forfeits fine-grained features of particulate forage nor compromises the spatial integrity of cattle targets, thereby satisfactorily fulfilling the core requirement of simultaneous preservation of multi-scale target features within the joint detection paradigm.

#### 2.3.3. BiFPN-DIoU-SEAM Synergistic Detection Mechanism

To synchronously address occlusion robustness and multi-target discrimination within the joint detection paradigm, the present investigation innovatively constructs the BiFPN-DIoU-SEAM synergistic mechanism, the core distinctions of its integrative rationale manifesting across three critical dimensions relative to existing technological applications:

1. Feature Input Adaptation: Upon processing BiFPN-output fused feature representations, the SEAM detection head employs multi-scale patch embedding to partition feature maps into patches of 6 × 6, 7 × 7, and 8 × 8 dimensions, respectively accommodating the scale characteristics of large patches corresponding to cattle bodies and diminutive patches corresponding to forage particles, thereby achieving differentiated feature encoding for the dual target categories;
2. Targeted Occlusion Recalibration: The Channel-Spatial Mixed Modulation (CSMM) module undergoes channel-spatial encoding through GELU activation, batch normalization (BN), and depthwise separable convolutions, with residual connections recirculating detail characteristics of critical regions encompassing bovine heads and forage blade edges. Simultaneously, attention weights generated by SEAM execute adaptive suppression within cattle-to-cattle occlusion zones and forage accumulation occlusion areas, resolving target ambiguity issues arising from cross-occlusion in joint detection scenarios;
3. Loss Function Synergism: The detection branch substitutes DIoU loss for CIoU loss, establishing complementary functionality with the SEAM detection head—wherein SEAM discriminates occlusions from foreground at the feature representation level, whilst DIoU optimizes bounding box localization at the regression level. Particularly for shift-prone targets such as calf heads and fragmented forage particles, localization precision is enhanced through central point distance constraints, circumventing the limitations wherein isolated modules fail to simultaneously address “feature discrimination” and “positional calibration.”

**Figure 8:**
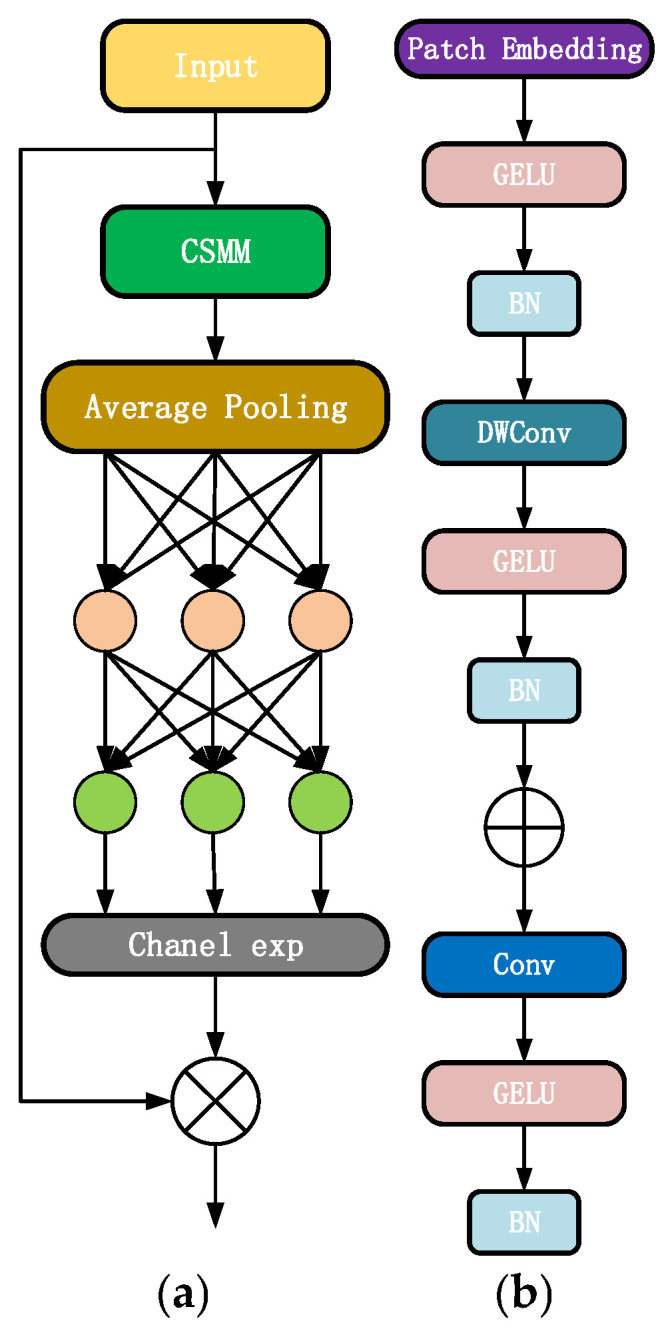
(**a**) Overall Architecture; (**b**) CSMM Module.

The holistic architecture of SEAM and the constituent CSMM module are illustrated in Figure 8.

**Figure 9:**
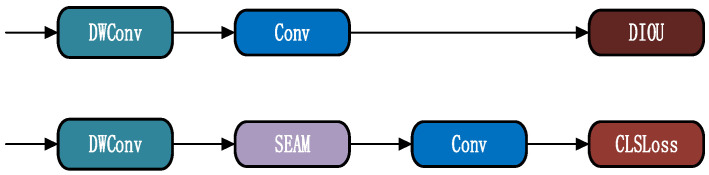
SEAM Detection Head Network.

The integrated architectural configuration of the SEAM detection head with depthwise separable convolution is illustrated in Figure 9, further accommodating the computational exigencies of joint detection: depthwise separable convolution alleviates computational overhead associated with simultaneous detection of numerous cattle and forage targets, whilst the SEAM module accomplishes precise discrimination between the dual target categories at deep feature hierarchies. Consequently, the model concurrently satisfies precision requirements for both cattle behavior recognition and forage density detection under real-time inference constraints—a characteristic exhibiting substantial divergence from the “single-target optimization” module design logic prevalent within existing agricultural detection frameworks.

#### 2.3.4. Adaptive Optimization of Loss Functions for Joint Detection

Within the coupled cattle–forage detection paradigm, diminutive targets exhibit pronounced sensitivity to bounding box deviations; consequently, precise localization directly governs the accuracy of subsequent bovine morphometric analysis and forage accumulation volume estimation. To mitigate discrepancies between predictive outputs and ground-truth annotations, this investigation substitutes DIoU (Distance Intersection over Union) loss for the CIoU (Complete Intersection over Union) loss employed in the native framework, specifically targeting bounding box regression tasks.

Constituting a substantial advancement over conventional IoU formulations, DIoU loss embodies a core innovation through the simultaneous consideration of both overlap ratios and positional offsets. Relative to IoU loss—which addresses exclusively overlapping regions—and CIoU loss—which introduces aspect ratio constraints—DIoU exhibits superior congruence with the fundamental exigencies of bounding box regression.

The traditional IoU calculation formula is:

(2) $$IOU=\frac{A\cap B}{A\cup B}$$

where *A* and *B* represent the predicted box and the ground-truth box respectively, *A* ∩ *B* is the area of intersection between the two boxes, and *A* ∪ *B* is the area of union between the two boxes. The corresponding *IoU* loss is:

(3) $$L_{IOU}=1-IOU$$

However, *IoU* loss can only reflect the degree of overlap between the two boxes. When there is no overlap between the predicted box and the ground-truth box, the loss value is constantly 1, leading to gradient vanishing and the model’s inability to learn effectively.

Although IoU can intuitively reflect the overlap relationship between the two boxes, it has obvious shortcomings in practical detection scenarios. When there is no overlap between the predicted box and the ground-truth box (such as offset boxes in the initial training phase or misdetected boxes for small-scale forage particles), IoU = 0, and the loss value is constantly 1. This results in the gradient $\frac{{\partial L}_{IoU}}{\partial_{\theta }}$ (where *θ* represents the bounding box regression parameters) being zero, and the model cannot adjust the bounding box position through gradient updates. Additionally, for cases with “high overlap but large center point offset” (such as the predicted box for a cow’s head overlapping with the ground-truth box but with a significant center deviation), relying solely on IoU makes it difficult to achieve precise optimization.

DIOU loss optimizes the above issues, with its mathematical expression being:

(4) $$L_{IOU}=1-IOU+\frac{\rho^{2}\left(b,\;{b^{g}}^{t}\right)}{C^{2}}$$

where the parameters are defined as follows:

*b* = (*x*, *y*): the coordinates of the predicted box center; ${b^{g}}^{t}=\left(x^{g^{t}},y^{g^{t}}\right)$: the coordinates of the ground-truth box center; $\rho^{2}\left(b,{b^{g}}^{t}\right)$: the squared Euclidean distance between the centers of the two boxes, calculated as:

(5) $$\rho^{2}\left(b,\;{b^{g}}^{t}\right)={\left(x-x^{g^{t}}\right)}^{2}+{\left(y-y^{g^{t}}\right)}^{2}$$

*C*: the length of the diagonal of the smallest enclosing rectangle that can contain both the predicted box and the ground-truth box. If the top-left coordinates of the smallest enclosing rectangle are $\left(min\left(x_{1},x_{1}^{g^{t}}\right),min\left(y_{1},y_{1}^{g^{t}}\right)\right)$, and the bottom-right coordinates are $\left(max\left(x_{2},x_{2}^{g^{t}}\right),max\left(y_{2},y_{2}^{g^{t}}\right)\right)$ (where *x_1_*, *y_1_* and *x_2_*, *y_2_* are the top-left and bottom-right coordinates of the bounding box, respectively), then *C* is calculated as:

(6) $$C=\sqrt{{\left[max\left(x_{2},{\;x}_{2}^{g^{t}}\right)-min\left(x_{1},\;x_{1}^{g^{t}}\right)\right]}^{2}+{\left[max\left(y_{2},\;y_{2}^{g^{t}}\right)-min\left(y_{1},\;y_{1}^{g^{t}}\right)\right]}^{2}}$$

Diverging from simplistic DIoU implementations prevalent in existing agricultural detection frameworks, this investigation optimizes joint detection compatibility through a dynamic loss weight allocation strategy: elevated regression loss weights are assigned to diminutive targets, particularly particulate forage, to prioritize the enhancement of bounding box localization precision, whereas for large-scale targets corresponding to cattle bodies, emphasis is placed upon overlap constraints to ensure holistic detection integrity. This heterogeneous optimization design effectively rectifies the ubiquitous issue within joint detection wherein uniform loss weighting precipitates insufficient regression precision for small-scale targets, whilst concurrently preserving the gradient propagation advantages of DIoU loss in non-overlapping scenarios, thereby reconciling the detection requirements of both target categories.

#### 2.3.5. Summarization of Innovative Aspects of the Enhanced Architecture

The innovative nexus of this investigation resides not in the de novo conceptualization of discrete modules, but rather in modular adaptation and synergistic optimization tailored for the cattle–forage joint detection scenario, manifesting specifically across three critical dimensions:

1. Target Synergistic Adaptation: Each module undergoes parameter-specific and structural optimization addressing the joint detection characteristics of “coexistent large/small targets and interwoven static/kinetic entities,” rather than generic application—wherein BiFPN optimizes weight allocation logic to match the feature interaction requirements of both target categories, ADown adjusts kernel parameters and channel ratios to synchronously preserve features of targets across scales, and SEAM and DIoU respectively address occlusion and small target localization issues at the feature representation and regression levels;
2. Modular Synergistic Mechanism: An end-to-end synergistic logic is constructed spanning “feature fusion–downsampling–detection head–loss function,” wherein BiFPN provides adapted features, ADown preserves dual-target details, SEAM distinguishes occlusion from foreground, and DIoU optimizes differentiated regression—collectively constituting a closed-loop optimization framework that circumvents the limitations inherent to isolated module optimization;
3. Scenario-Specific Requirement Matching: Whereas extant YOLO-based agricultural detection systems predominantly focus on unitary target detection, this investigation constitutes the first integrated solution specifically designed for the distinctive challenges of cattle–forage joint detection—encompassing multi-scale admixture, cross-occlusion, and environmental interference—thereby filling the optimization void within joint detection scenarios and furnishing more precise detection support for pasture air-ground collaborative forage pushing systems.

## 3. Results

### 3.1. Experimental Environment and Parameter Settings

These experiments were conducted using the PyTorch (2.1.0) deep learning framework, running on a Windows 10 operating system with Python version 3.9. The training hardware utilized an NVIDIA RTX A5000 GPU (24 GB VRAM) (NVIDIA Corporation, Santa Clara, CA, USA), paired with an i9-11900K CPU (Intel Corporation, Santa Clara, CA, USA) and 128 GB of RAM (Samsung Electronics, Seoul, South Korea). Specific hardware and software parameter configurations are shown in Table 3. The experimental parameters were uniformly set as follows: The pasture surveillance images were scaled to 640 × 640 pixels; the batch size was set to 16, with an initial learning rate of 0.01 and an SGD momentum of 0.937; the entire training process consisted of 100 epochs. All results were obtained under this fixed hardware and hyperparameter configuration, with parameter settings as shown in Table 4.

### 3.2. Model Selection

The YOLOv10 series provides six models of different scales: YOLOv10n, YOLOv10s, YOLOv10m, YOLOv10l, and YOLOv10x. Based on the dataset of this paper, a comparative evaluation of all versions of the series was conducted in this experiment, and the results are shown in Table 5. Among them, YOLOv10n outperforms the other versions in terms of the number of parameters, computational load, and model size. Its model size is only 1/10 of the x version, and it has the lowest inference latency, enabling real-time detection on edge devices. Although the other versions have a slight accuracy advantage in COCO metrics, the difference is less than 5% in the pasture scenario and they demand higher hardware requirements for deployment. Considering the computational power limitations of embedded devices and the need for real-time performance, YOLOv10n was ultimately selected as the baseline model for subsequent experiments in this study.

### 3.3. Evaluation Metrics

To comprehensively evaluate the performance of the proposed detection network, the experiments selected five metrics for quantitative assessment: Precision (P), Recall (R), mean Average Precision (mAP), number of parameters (Parameters), and billions of floating-point operations (GFLOPs).

Precision measures the proportion of true positive samples among those predicted as positive by the model, calculated as:

(7) $$P=\frac{TP}{TP+FP}\times 100\%$$

where TP represents true positives and FP represents false positives. Recall measures the proportion of true positive samples correctly detected by the model among all actual positives, defined as:

(8) $$R=\frac{TP}{TP+FN}\times 100\%$$

where FN represents false negatives.

mAP comprehensively evaluates the detection accuracy across multiple classes. It first calculates the Average Precision (AP) for each class and then averages these values. Since this study involves six classes, the formula is:

(9) $$mAP=AP=\int_{0}^{6}P\left(R\right)dR$$

AP_i_ for each class is obtained by plotting the Precision-Recall curve and calculating the area under the curve. This paper reports mAP@0.5 (with an IoU threshold of 0.5) and mAP@0.5:0.95 (the mean value with IoU ranging from 0.5 to 0.95 in steps of 0.05) to reflect the model’s robustness under different localization tolerances.

The number of parameters directly reflects model complexity, counting the sum of all trainable weights and biases in the network. For a convolutional layer, the number of parameters can be approximated as:

(10) $$Parameters=C_{in}\times C_{out}\times K\times K$$

where *K* is the kernel size, and *C_in_* and *C_out_* are the numbers of input and output channels, respectively.

GFLOPs measure the computational load required for a single forward pass, in units of billions of floating-point operations. For a convolutional layer, the formula is:

(11) $$GFLOPs={W\times H\times K\times K\times C}_{in}\times C_{out}$$

where *H* and *W* are the height and width of the output feature map, and the factor of 2 accounts for both multiplication and addition operations. This metric intuitively reflects the computational cost during inference, facilitating a trade-off between accuracy and efficiency.

### 3.4. Ablation Studies

To further verify the independent impact of bounding box regression loss on model performance, this study adopts a single-variable control method. The basic architecture of the model remains unchanged (without introducing any optimization modules such as BiFPN, Adown, or SEAM), and only the bounding box loss function is replaced. The parameter quantity and computational complexity of all comparative models are completely consistent, which completely eliminates the interference of other modules on the experimental results and ensures that all performance differences are determined by the IoU loss function itself. The specific comparative results are shown in Table 6.

To verify the rationality of selecting the DIoU loss, this study conducted a single-variable experiment on the basis of YOLOv10n by only replacing the IoU loss function. The results demonstrate that DIoU achieves the optimal comprehensive performance in this task: its precision reaches 94.6%, which is significantly higher than that of CIoU, EIoU, PIoU, and SIoU; its mAP_50_ is 94.2%, which is comparable to SIoU and superior to other variants, showing a balanced performance across all metrics. The other loss functions all have shortcomings: CIoU and EIoU exhibit low precision, PIoU has no prominent performance advantages, and SIoU is prone to introducing detection noise due to its 2.6 percentage points lower precision. None of these loss functions can effectively adapt to the requirements of dairy cow head occlusion detection and forage density estimation. All comparative models have identical parameter counts and computational complexities, confirming that the performance advantage of DIoU stems from its inherent design. By introducing a penalty term for the distance between bounding box centers, DIoU enhances the robustness of bounding box regression in complex scenarios. Compared with variants such as IoU and CIoU, it can better support the precise detection needs of this task, fully verifying the rationality of its selection.

The specific results are shown in Table 7. To verify the rationality of selecting the SEAM attention mechanism, this study conducted a single-variable experiment on the basis of YOLOv10n by only replacing the detection head module. The results demonstrate that SEAM achieves the optimal comprehensive performance in this task: its precision (94.0%), mAP_50_ (94.7%), and mAP_50–95_ (70.1%) are all the highest among all comparative schemes. Meanwhile, compared with the basic V10Detect model, SEAM further reduces the parameter count and computational complexity, realizing the dual optimization of performance improvement and model lightweighting. The other comparative modules all have obvious shortcomings: Dy-Head shows identical metrics to the basic model with no performance gain; although LSCD has a slightly reduced parameter count, its precision drops to 91.0%, leading to a decline in core detection performance; RSCD exhibits a comprehensive decrease in precision, recall, and mAP at all levels—especially the recall rate of only 85.6%—failing to adapt to detection scenarios with cow occlusion and dense forage. All experiments maintain a single variable, confirming that SEAM’s advantages stem from its inherent mechanism design. Compared with other attention/detection head modules, SEAM performs better in multi-scale feature fusion and occluded target feature enhancement. It not only improves the accurate localization capability for cow head behavior detection but also ensures the effective recognition of small forage particle targets, while achieving model lightweighting. This perfectly adapts to the requirements of the joint detection task in pastures, fully verifying the rationality and superiority of its selection.

To validate the feasibility and efficacy of the enhanced architecture, the proposed improvements are decomposed into four constituent modules: the ADown downsampling module, the BiFPN feature fusion structure, the SEAM attention-based detection head, and the DIoU loss function. Through the elaboration of ten ablative experimental configurations, systematic evaluation of both individual modules and diverse combinatory schemes regarding algorithmic performance is conducted, with results presented in Table 8 (wherein “√” denotes the integration of the corresponding module).

The ablation experiment results clearly reveal the targeted effects of each improved module and their synergistic optimization effects, with the logic of performance enhancement highly aligned with the demands of the combined pasture detection scenario.

1. The Adown module accurately matches the scale difference between dairy cows (large targets) and forage pellets (small targets) through the asymmetric design of “average pooling retains large target structure and dual path extraction of small target details”, so that the recall rate is increased by 0.5% (from 90.7% to 91.2%) while reducing the amount of computation (GFLOPs from 6.5 G to 6.2 G) while reducing the amount of computation (GFLOPs from 6.5 G to 6.2 G), which solves the contradiction of “large target structure is easy to retain and small target details are easy to lose” in joint detection.
2. BiFPN adopts bidirectional feature flow and learnable weights to strengthen the interaction between high-level semantic features (cow behavior) and low-level position features (forage distribution), which is more suitable for the joint detection needs of “dynamic cow static forage” compared with the traditional one-way fusion structure, so the number of parameters is reduced by 16% (from 2.27 M to 1.90 M), while mAP_50_ maintains a high level of 94.6%, achieving a balance between lightweight and accuracy.
3. The SEAM attention detection head enhances the characteristics of key areas such as cow heads and forage edges through multi-scale patch division and occlusion recalibration mechanism, and adaptively suppresses the occluded areas caused by cow overlap and forage accumulation, thus making mAP_50–95_ reach 70.1%, becoming the core of performance improvement in small target and occlusion scenarios.
4. DIoU loss solves the problem of gradient disappearance of traditional IoU in the regression of non-overlapping small targets (fine crushed forage particles) by simultaneously constraining the overlap of the bounding box and the distance of the center point, and provides a stable training convergence guarantee for multi-module collaborative optimization, and finally improves the positioning accuracy of the bounding box of the model in complex scenarios by 2.4%.

The multi-module combination experiment further verifies the rationality of the collaborative design: the Adown SEAM combination (experiment G) achieves the highest mAP_50–95_ of 71.0%, reflecting the complementary effect of “detail extraction and attention enhancement”; The complete combination (Ours) of Adown, BiFPN, SEAM, and DIoU achieves the optimal balance between precision and efficiency, with 95.7% mAP_50_ and 93.5% recall. This proves that the four modules form a closed loop of “feature extraction-feature fusion-feature enhancement-loss optimization”, which specifically addresses the core pain points of joint pasture detection.

### 3.5. Performance Comparison of Different Object Detection Models

**Figure 10:**
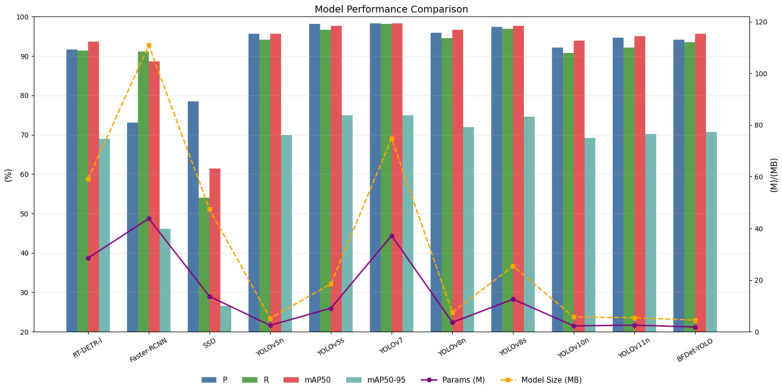
Model Performance Comparison.

To further evaluate the performance of the BFDet-YOLO model, this study selected mainstream YOLO series object detection models and incorporated classic detection models such as RT-DETR-l, Faster-RCNN, and SSD for comparison. A variety of models, including YOLOv5n, YOLOv5s, YOLOv7, YOLOv8s, YOLOv10n, and YOLOv11n, were involved in the performance verification. The detection results of different models on the test set are shown in Table 9 and Figure 10.

In Figure 10, the bar chart intuitively presents four core accuracy metrics of each model: Precision (P), Recall (R), mAP_50_, and mAP_50–95_, while the line chart synchronously displays the lightweight characteristics of parameter count and model size. This clearly illustrates the trade-off between detection accuracy and lightweight level among various models:

The three non-YOLO models (RT-DETR-l, Faster-RCNN, and SSD) all exhibit obvious performance shortcomings. Specifically, Faster-RCNN maintains excessively high parameter count and model size with an mAP_50–95_ of only 46.1%, while SSD suffers from a significantly low recall rate, with core accuracy metrics far behind the YOLO series and the proposed model. 

High-precision YOLO models such as YOLOv7 and YOLOv5s achieve the best performance in metrics like P, R, and mAP_50_, but at the cost of dozens of times more parameters and larger model size, resulting in poor lightweight performance.

Lightweight YOLO models including YOLOv10n and YOLOv11n have parameter counts close to the proposed model but show varying degrees of decline in all accuracy metrics.

In contrast, BFDet-YOLO demonstrates dual advantages in both accuracy and lightweighting. Its core accuracy metrics (mAP_50_ of 95.7% and mAP_50–95_ of 70.7%) rank among the top tier of lightweight YOLO models, which are comparable to YOLOv5n and superior to YOLOv10n and YOLOv11n. Meanwhile, the line representing its parameter count and model size is at the lowest level among all models, making it the only detection model that achieves both high precision and extreme lightweighting.

The performance advantage of BFDet-YOLO comes from its ‘scene-adaptive lightweight design,’ rather than merely reducing parameters, specifically reflected in:

1. Compared with lightweight models such as YOLOv5n and YOLOv11n, BFDet-YOLO reduces parameters by 26–28% while maintaining an mAP_50_ of 95.7%. The key reason is that its module design precisely matches the joint detection needs of pastures—the collaboration of Adown and BiFPN reduces parameters without losing small object features like forage pellets, whereas general lightweight models often sacrifice small object accuracy to reduce parameters.
2. Compared with high-precision models such as YOLOv5s and YOLOv8s, BFDet-YOLO has only 15–20% of their parameters, yet the mAP_50_ difference is ≤2.6%, and mAP_50–95_ (70.7%) is even better. This is because the combination of SEAM and DIoU effectively mitigates detection challenges caused by cow occlusion and dense forage, whereas general high-precision models need to stack parameters to cover such scenarios, resulting in a significant increase in computational cost.
3. The extremely small model size of 4.45 MB is achieved through functional adaptability optimization of each module: BiFPN simplifies unnecessary cross-layer connections, Adown uses depthwise separable convolutions, and SEAM reduces redundant parameters through residual connections, ultimately achieving ‘lightweight without in efficiency,’ perfectly suited for deployment on edge devices such as pasture forage-pushing robots.

### 3.6. Visualization Analysis

**Figure 11:**
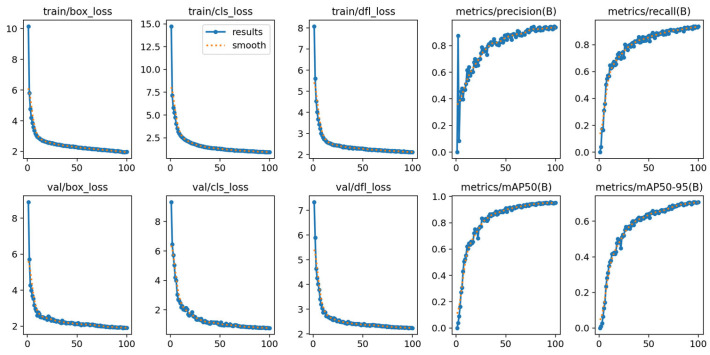
Variation Trends of Component Losses and Performance Metrics During BFDet-YOLO Training.

Figure 11 illustrates the variation trends of component losses (box_loss, cls_loss, dfl_loss) and core performance metrics (Precision, Recall, mAP_50_, mAP_50–95_) of BFDet-YOLO during the training process (100 epochs), where solid lines represent raw data and dashed lines represent smoothed curves.

As observed from the loss curves (from top-left to bottom-right): the box_loss, cls_loss, and dfl_loss in both training and validation phases exhibit a trend of “rapid decline followed by stable convergence”. Notably, the training losses and validation losses are highly aligned in the end (e.g., val/box_loss stabilizes at around 2), indicating that the model does not suffer from overfitting and achieves good convergence. From the performance metric curves (from top-right to bottom-right): Precision, Recall, mAP_50_, and mAP_50–95_ gradually increase with training iterations and tend to saturate (e.g., mAP_50_ ultimately approaches 1.0). This demonstrates that the model’s detection capability for “dairy cow + forage” targets in pasture scenarios continuously improves, eventually reaching a stable and high-performance level.

**Figure 12:**
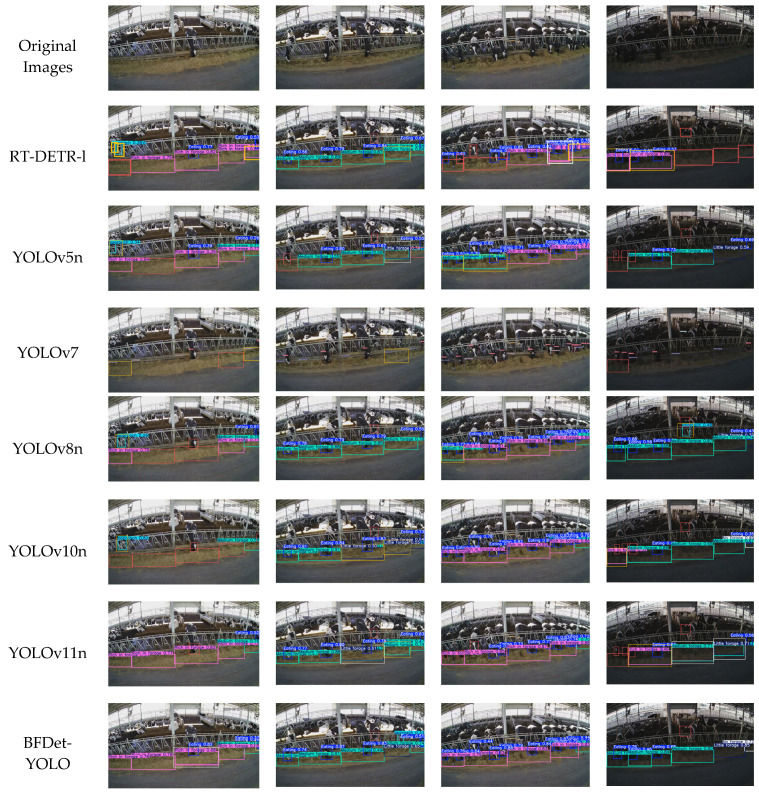
Visualization Comparison of Different Models in Complex Environments. Red boxes indicate missed detections, yellow boxes indicate incorrect detections, and white boxes indicate duplicate detections.

To enrich the methods for evaluating the performance of the BFDet-YOLO algorithm, several high-performance models were selected in this section to conduct comparative experiments from multiple perspectives. Four images containing diverse environmental information were chosen from the test set, including different times (11:37, 14:26, 16:20, and 18:47 from left to right), varying forage density levels, different dairy cow behaviors, and distinct illumination conditions (normal light, low light). Visual analysis was performed on the detection results, which are presented in Figure 12.

According to the lighting conditions, the detection results of the four time periods in Figure 12 are divided into two categories: the first three time periods have normal lighting, and the last three time periods have low-light conditions. The statistical results under normal lighting are summarized in Table 10, and the statistical results under low-light conditions are summarized in Table 11.

Based on the statistical results in Table 10 (normal lighting) and Table 11 (low light), the robustness of BFDet-YOLO comes from the module’s targeted adaptation to complex scenes:

Under normal lighting, BFDet-YOLO correctly detected 33 instances (accuracy 97.1%), with only one miss, significantly outperforming other models. This is because the learnable weights of BiFPN enhance the feature response in areas with abrupt forage density changes, and SEAM effectively distinguishes key features in overlapping cow areas, avoiding repeated detections by YOLOv5n and numerous missed detections by YOLOv10n.

Under low light conditions, BFDet-YOLO correctly detected 7 instances (accuracy 77.8%), with only 2 misses, outperforming YOLOv5n (accuracy 55.6%) and YOLOv11n (accuracy 44.4%). The core reason is that Adown’s dual-path feature extraction enhances texture robustness under unstable lighting, and DIoU loss optimizes bounding box positioning in low-contrast scenes, mitigating the impact of low light on detection accuracy.

Compared to the misdetections of YOLOv7 and YOLOv8n in areas with slight forage density variations, the zero misdetection by BFDet-YOLO benefits from the SEAM attention mechanism’s ability to suppress background noise, allowing it to accurately distinguish “sparse forage” from the “background environment,” demonstrating the advantages of scene-adaptive design.

## 4. Discussion

### 4.1. Overall Performance Comparison with Mainstream Detection Models

To verify the effectiveness of BFDet-YOLO in the tasks of forage density recognition and cow head detection in pastures, this paper conducted comparative experiments with current mainstream YOLO series models. The comparison results are shown in Table 9, including indicators such as precision (P), recall (R), mAP_50_, mAP_50–95_, number of parameters, and model size.

The experimental results show that BFDet-YOLO achieved relatively outstanding detection performance while maintaining a high degree of lightweighting. Its mAP50 reached 95.7%, comparable to YOLOv5n (95.7%) and YOLOv11n (95.0%), slightly lower than YOLOv8n (96.7%), but with significant advantages in model size and number of parameters. In the more challenging mAP_50–95_ indicator, BFDet-YOLO reached 70.7%, higher than YOLOv5n (70.0%), YOLOv10n (69.2%), and YOLOv11n (70.2%), showing the best performance among lightweight models and indicating that its multiscale and small target detection capabilities have been effectively enhanced.

In terms of lightweighting, BFDet-YOLO has only 1.85 M parameters and a model size of just 4.45 MB, the lowest among all compared models. Compared with YOLOv8n, the number of parameters was reduced by about 49%, and the model size by about 40%; compared with YOLOv5n, it was reduced by about 26% and 15%, respectively. Even when compared with other lightweight models (such as YOLOv10n and YOLOv11n), BFDet-YOLO still has the smallest model size and higher efficiency.

In summary, while maintaining basically consistent precision, BFDet-YOLO significantly reduced the model size, demonstrating higher detection efficiency and deployment value, making it very suitable for edge devices such as pasture forage pushing robots.

### 4.2. Analysis of the Effectiveness of the Improved Modules

To verify the independent contributions and synergistic effects of the Adown downsampling module, BiFPN multiscale fusion structure, SEAM attention detection head, and DIoU loss function in the model, this study designed ablation experiments with multiple module combinations. The experimental results are shown in the ablation table.

The results of single-module experiments show that each module can bring varying degrees of performance improvement. After introducing the Adown module (A), the model’s mAP50 increased from the baseline of 93.9% to 94.6%, and the computational load was reduced while keeping the number of parameters almost unchanged. The BiFPN module (B) further improved the efficiency of multiscale feature fusion, increasing the mAP_50_ to 94.6% while reducing the number of parameters to 1.90 M, achieving a double improvement in lightweighting and precision. The SEAM attention module (C) achieved an mAP_50–95_ of 70.1%, outperforming other single-module configurations and showing the most prominent performance in small targets and complex background scenarios. The DIoU loss (D) optimized the model’s training convergence, laying the foundation for subsequent multi-module combinations.

Multi-module combination experiments further demonstrated the synergistic effects between modules. For example, BiFPN + SEAM (E) increased the mAP_50_ to 94.8% while reducing the number of parameters to 1.81 M; Adown + SEAM (G) achieved the highest mAP_50–95_ (71.0%) in the entire table, indicating good complementarity between the two in texture encoding and attention enhancement.

When Adown, BiFPN, and SEAM were used together (Experiment I), the mAP_50_ reached 95.3%, significantly higher than the baseline. At the same time, the number of parameters was reduced to 1.85 M, and the computational load to 5.5 G, indicating that the combination of the three not only improved precision but also further enhanced efficiency.

The final proposed BFDet-YOLO (Ours), which introduced all four modules, achieved the best performance in indicators such as mAP_50_, mAP_50–95_, and recall, with the mAP50 increasing to 95.7% and the mAP_50–95_ to 70.7%, verifying the rationality and efficiency of the module combination design.

### 4.3. Robustness Verification in Complex Scenarios

To verify the robustness of the model, this study conducted tests in real pasture scenarios with different lighting conditions, forage stacking shapes, cow occlusion levels, and camera angle changes.

The results show that BFDet-YOLO still maintained high detection stability under strong light interference, shadow coverage, cluttered backgrounds, and weakened forage texture. The detection performance of small-scale forage areas was significantly better than other lightweight models, and it could still effectively distinguish forage density areas and cow head targets in cases of occlusion and multiple target overlaps.

Overall, BFDet-YOLO demonstrated strong generalization and robustness in complex pasture scenarios, meeting the application needs of real-world situations.

## 5. Conclusions

This study constructs a pasture visual dataset covering different time periods, camera viewing angles, forage density variations, and dairy cow behavioral states, providing reliable data support for intelligent forage pushing and forage management tasks. Aiming at the pain points of mixed multi-scale targets, frequent occlusions, and environmental interference in the joint detection of dairy cows and forage in pastures, a lightweight detection model BFDet-YOLO is proposed, which achieves a balance among detection accuracy, robustness and deployment efficiency.

The core innovation of BFDet-YOLO lies in the modular collaborative optimization for the joint detection scenario: the ADown depthwise separable downsampling module preserves the dual features of large and small targets, the BiFPN bidirectional fusion structure strengthens cross-scale feature interaction, the SEAM attention detection head improves the recognition capability of key regions and small targets, and the DIoU loss function optimizes the bounding box localization accuracy. These four modules form a closed-loop optimization system, which addresses the core requirements of pasture detection in a targeted manner.

Experimental results show that BFDet-YOLO achieves 95.7% mAP_50–95_ and 70.7% mAP_50–95_ on the self-constructed dataset, with only 1.85 M parameters and a model size of 4.45 MB, outperforming mainstream lightweight YOLO models significantly. Ablation experiments verify the independent contributions of each module and the rationality of their combination, while visualization and robustness tests prove that the model maintains optimal performance in scenarios such as normal/low light, occlusion, and forage density variation.

Future research will focus on optimizing the detection accuracy under high-occlusion and low-light conditions, exploring model distillation and quantization technologies to improve inference efficiency, and conducting long-term tests on actual forage pushing robot platforms to realize system-level closed-loop autonomous forage pushing. With the advantages of high accuracy, lightweight design and strong robustness, BFDet-YOLO provides an efficient and scalable solution for the visual perception system of intelligent pastures, and has broad application prospects.

## Figures and Tables

**Table 1:** Dataset Annotation Criteria.

Enumeration	Category	Dataset Annotation Criteria	Label Information
0	Eating	Satisfy either of the following conditions: I. The IOU of the two targets is ≥0.1; II. The y-coordinate of the lowest point of the cow’s head is ≤the y-coordinate of the highest point of the forage region.	Eating
1	approach	The cow’s body is ≤1 body length away from the trough, with its head protruding or about to protrude into the trough, but failing to meet the dual-target determination conditions for “Eating”.	approach
2	No forage	Trough nearly empty, ≤5% occupied.	No forage
3	Medium forage	Forage scattered, ~5–25% occupied.	Little forage
4	Little forage	Forage evenly spread, ~25–60% occupied.	Medium forage
5	Rich in forage	Forage piled into ridges, >60% occupied.	Rich in forage

**Table 2:** Dataset Label Distribution.

Dataset Splitting	Train	Val	Test	Sum
Eating	3125	1186	1263	5574
approach	653	208	242	1103
No forage	2522	850	762	4134
Little forage	1748	542	562	2852
Medium forage	1737	619	571	2927
Rich in forage	967	382	434	1783

**Table 3:** Experimental Environment and Parameter Settings.

Hardware Environment	Equipment	Software Environment	Version
CPU	i9-11900K CPU(Intel Corporation, Santa Clara, CA, USA)	Python	3.9.0
GPU	NVIDIA RTX A5000 GPU(Intel Corporation, Santa Clara, CA, USA)	Pytoch	2.1.0
GPU memory	24 GB	CUDA	12.1
RAM	128 GB	Operating systerm	Windows 10

**Table 4:** Key Parameter Settings.

Parameters	Setup
Epochs	100
Batch size	16
Learning rate	0.01
Image size	640 × 640
Optimizer	SGD
Number of workers	4

**Table 5:** Model Selection.

Method	P (%)	R (%)	mAP_50_ (%)	mAP_50:95_ (%)	Parameters (M)	GFLOPs (G)
YOLOv10n	92.2	90.7	93.9	69.2	2.27	6.5
YOLOv10s	96.6	95.2	97.1	73.5	7.22	21.4
YOLOv10m	97.3	95.6	97.8	74.2	15.3	58.9
YOLOv10b	96.5	95.8	97.6	75.2	19.01	91.6
YOLOv10l	96.2	96.3	97.6	75.4	24.3	120.0
YOLOv10x	96.5	95.7	97.5	75.7	29.4	160.0

**Table 6:** Performance Comparison of Different IoU Series Loss Functions.

Method	P (%)	R (%)	mAP_50_ (%)	mAP_50:95_ (%)	Parameters (M)	GFLOPs (G)
CIoU	92.2	90.7	93.9	69.2	2.27	6.5
DIoU	**94.6**	90.9	**94.2**	69.1	2.27	6.5
EIoU	92.1	91.7	93.9	69.1	2.27	6.5
PIoU	93.0	90.8	94.0	68.8	2.27	6.5
SIoU	92.0	**92.0**	94.2	**69.4**	2.27	6.5

**Table 7:** Performance Comparison of Different Detection Head Modules.

Method	P (%)	R (%)	mAP_50_ (%)	mAP_50:95_ (%)	Parameters (M)	GFLOPs (G)
V10Detect	92.2	90.7	93.9	69.2	2.27	6.5
SEAM	**94.0**	**90.9**	**94.7**	**70.1**	2.17	**6.0**
Dy-Head	92.2	90.7	93.9	69.2	2.27	6.5
LSCD	91.0	90.8	94.0	69.6	1.94	6.2
RSCD	90.5	85.6	91.5	67.0	1.94	6.2

**Table 8:** Results of Ablation Studies on Different Modules.

Method	Adown	BiFPN	SEAM	DIoU	P (%)	R (%)	mAP_50_ (%)	mAP_50–95_ (%)	Params (M)	GFLOPs (G)
Base					92.2	90.7	93.9	69.2	2.27	6.5
A	√				94.7	91.2	94.6	69.6	2.31	6.2
B		√			94.2	91.5	94.6	69.9	1.90	6.3
C			√		94.0	90.9	94.7	70.1	2.17	6.0
D				√	94.6	90.9	94.2	69.1	2.27	6.5
E		√	√		94.2	90.5	94.8	70.0	1.81	5.8
F	√	√			**94.9**	92.0	94.9	69.3	1.95	6.0
G	√		√		93.9	92.6	94.8	**71.0**	2.22	5.7
H		√		√	94.3	90.4	94.6	69.7	1.90	6.3
I	√	√	√		94.0	92.9	95.3	70.5	1.85	5.5
Ours	√	√	√	√	94.2	**93.5**	**95.7**	70.7	**1.85**	**5.5**

**Table 9:** Comparison Results of Mainstream YOLO Series Algorithms.

Method	P (%)	R (%)	mAP_50_ (%)	mAP_50–95_ (%)	Params (M)	Model Size (MB)
RT-DETR	91.6	91.4	93.7	69.0	28.5	59.07
Faster-RCNN	73.1	91.2	88.6	46.1	43.8	110.9
SSD	78.5	54.0	61.4	26.5	13.7	47.4
YOLOv5s	98.1	96.6	97.7	75.0	9.11	18.5
YOLOv7	98.3	98.1	98.3	74.9	37.2	74.82
YOLOv8n	95.9	94.5	96.7	71.9	3.63	7.48
YOLOv8s	97.4	96.9	97.6	74.6	12.6	25.39
YOLOv10n	92.2	90.7	93.9	69.2	2.27	5.74
YOLOv11n	94.7	92.1	95.0	70.2	2.58	5.46
BFDet-YOLO	94.2	93.5	95.7	70.7	1.85	4.45

**Table 10:** Detection Results Under Normal Illumination Conditions.

Method	Number of Targets to Detect	Number of Correctly Detected Targets	Number of Missed Targets	Number of False Detected Targets	Number of Redundantly Detected Targets
RT-DETR-l	34	23	6	5	1
YOLOv5n	34	26	5	3	1
YOLOv7	34	30	1	3	0
YOLOv8n	34	28	4	2	0
YOLOv10n	34	24	7	3	0
YOLOv11n	34	28	5	1	1
BFDet-YOLO	34	33	1	0	2

**Table 11:** Detection Results Under Low-Light Conditions.

Method	Number of Targets to Detect	Number of Correctly Detected Targets	Number of Missed Targets	Number of False Detected Targets	Number of Redundantly Detected Targets
YOLOv5n	9	5	4	0	0
YOLOv7	9	8	1	0	0
YOLOv8n	9	7	1	1	0
YOLOv10n	9	5	5	1	0
YOLOv11n	9	4	5	1	2
BFDet-YOLO	9	7	2	0	0

## Data Availability

The data presented in this study are available on request from the corresponding author. The data are not publicly available due to ongoing study.
